# Account of a Fact Relative to Menstruation, Not Hitherto Described

**Published:** 1791

**Authors:** Thomas Denman

**Affiliations:** Licentiate in Midwifery of the Royal College of Physicians, London.


XII. *Account of a Fact relative to Menstruation, not hitherto described. Communicated in a Letter to Dr.* Simmons *by* Thomas Denman, *M. D. Licentiate in Midwifery of the Royal College of Physicians, London.*

*To Dr.* Simmons.

Dear Sir,

ALL the common circumstances attending menstruation have been well described by various authors; but having frequently seen a substance expelled with the menstrual discharge, which had escaped observation, I beg leave, by your favour, to give a short account of it.

In the examination of that discharge, for the purpose of investigating the state of the uterus, a membranous substance had been observed, which passed, without any particular notice, as the token of an early conception, or as the casual form of coagulated blood. Examining this substance with greater attention, I constantly found that one surface had a flocky appearance, appearance, and the other a perfectly ſmooth one; that it had, in all reſpects, the reſemblance of that membrane which Ruyſch had called the *villous*, and which the late Dr. Hunter, ſpeaking of abortions, had deſcribed with great preciſion, and called the *decidua*. To put the matter out of doubt, about two years ago I requeſted the favour of Dr. Baillie to examine ſome portions of it, and he agreed with me in thinking it ſimilar to the *decidua*.

Having never obſerved this membrane diſcharged by unmarried women, a doubt aroſe whether it was not really a conſequence of an early conception; but I have the moſt undoubted proofs that it may be formed without connubial communication, and that the uterus, in ſome women, has the property of forming it in the interval between, or at each period of the menſtrual diſcharges. It ſeems particularly neceſſary to eſtabliſh this fact, as the appearance of the membrane might give riſe to erroneous opinions and unjuſt reflections.

In every caſe, in which this membrane has been obſerved, the women have menſtruated with pain, and the diſcharge has flowed ſlowly, and apparently with difficulty, till the membrane brane has come away, which in ſome caſes has been in ſmall flakes, and in others in pieces equal to half the extent of the cavity of the uterus, of which they retained the ſhape. But whether this membrane be expelled in every caſe of painful menſtruation, my experience does not enable me to decide.

No woman, in the habit of expelling this membrane, has been known to conceive; and this obſervation leads me to ſpeak of the treatment which has been adviſed for making ſuch a change in the ſtate of the uterus, that it ſhould be diveſted of the property of forming this membrane at the time of menſtruation.

There does not appear to be any external peculiarity of conſtitution, or diſpoſition to any other complaint in thoſe who have been liable to the formation of this membrane, which ſeems to be a proper office performed at an improper time. Recourſe has been generally had to mercurial medicines in one form or other, ſometimes as active purges, and ſometimes carried ſo far as to occaſion a ſlight ſalivation: but the ſafeſt method, and, as far as I know, the moſt effectual, is, to give ſmall doſes of calomel every night at bed time for ſeveral weeks together, and and twice, in the courfe of the day, a large dofe of the volatile tincture of bark.

I remain, dear SIR,

Your very humble Servant,

THOMAS DENMAN.

*Old Burlington Street,*

May 5, 1791.

